# A Dose-Dependent Effect of Carnipure^®^ Tartrate Supplementation on Endurance Capacity, Recovery, and Body Composition in an Exercise Rat Model

**DOI:** 10.3390/nu12051519

**Published:** 2020-05-23

**Authors:** Kazim Sahin, Cemal Orhan, Osman Kucuk, Nurhan Sahin, Mehmet Tuzcu, Besir Er, Shane Durkee, Aouatef Bellamine

**Affiliations:** 1 Department of Animal Nutrition, Faculty of Veterinary Medicine, Firat University, 23119 Elazig, Turkey; nsahinkm@yahoo.com (K.S.); corhan@firat.edu.tr (C.O.); nsahin@firat.edu.tr (N.S.); 2 Department of Animal Nutrition, School of Veterinary Medicine, Erciyes University, 38280 Kayseri, Turkey; osmankucukwy@yahoo.com; 3 Department of Biology, Faculty of Science, Firat University, 23119 Elazig, Turkey; mtuzcu@firat.edu.tr (M.T.); beshir.er@hotmail.com (B.E.); 4 Lonza Consumer Health Inc., Morristown, NJ 07960, USA; shane.durkee@lonza.com

**Keywords:** L-carnitine, recovery, exercise, endurance, fatigue, body mass index, metabolic parameters

## Abstract

The objective of this work is to investigate the effects of Carnipure^®^ Tartrate (CT) supplementation with or without exercise on endurance capacity, recovery, and fatigue by assessing time to exhaustion as well as body weight and composition in rats. In addition, antioxidant capacity has been evaluated by measuring malondialdehyde (MDA) levels and antioxidant enzyme (superoxide dismutase, SOD; catalase, CAT; glutathioneperoxidase; GSHPx) activities. Fifty-six male Wistar rats were divided into eight groups including seven rats each. A control group did not receive CT nor exercise. Another control group received 200 mg/kg CT without exercise. The other six groups of rats went through an exercise regimen consisting of a 5-day training period with incremental exercise capacity, which was followed by 6 weeks of the run at 25 m/min for 45 min every day. CT was supplemented at 0, 25, 50, 100, 200, and 400 mg/kg per day during the 6 weeks. Rats submitted to exercise and supplemented with CT had a significant and dose-dependent increase in time to exhaustion and this effect seems to be independent of exercise (*p* < 0.05). Additionally, recovery and fatigue were improved, as shown by a significant and dose-dependent decrease in myoglobin and lactic acid plasma levels, which are two markers of muscle recovery. CT supplementation led to a dose-response decrease in body weight and visceral fat. These effects become significant at 200 and 400 mg/kg doses (*p* < 0.05). Additionally, the antioxidant capacity was improved, as shown by a significant and dose-dependent increase in SOD, CAT, and GSHPx. Serum MDA concentrations decreased in exercising rats with CT supplementation. CT supplementation led to a decrease in serum glucose, triglycerides, and total cholesterol concentrations with the lowest levels observed at 400 mg/kg dose (*p* < 0.05). These effects correlated with a significant dose-dependent increase in serum total L-carnitine, free L-carnitine, and acetyl-carnitine, which linked the observed efficacy to CT supplementation. These results demonstrate that CT supplementation during exercise provides benefits on exercise performance, recovery, and fatigue as well as improved the lipid profile and antioxidant capacity. The lowest dose leads to some of these effects seen in rats where 25 mg/kg corresponds to 250 mg/day as a human equivalent.

## 1. Introduction

The importance of physical training in promoting various health benefits, such as increased insulin sensitivity, diminished risk factors for metabolic syndrome, reduced blood pressure (preventing hypertension), and improved muscle metabolism and antioxidant capacity have been demonstrated [1,2,3,4,5,6,7,8]. Physical exercise promotes muscle mass and muscle functions by increasing muscle protein synthesis and stimulating mitochondrial biogenesis [9,10]. It has also been found that high-intensity endurance training increases energy expenditure and induces the secretion of lipolytic hormones to facilitate post-exercise energy expenditure [11,12].

Dietary supplements—such as L-carnitine—known to enhance performance during physical exercises have been widely used. International surveys revealed that every two out of three adult and adolescent elite track and field athletes participating in world-championship competitions took at least one dietary supplement [13]. L-carnitine is also suggested as a dietary supplement in recovery after exercise, as recently reviewed [14], in addition to its effect on fat metabolism [15]. This resulted in increased endurance capacity. In this respect, L-carnitine is an ergogenic substance for athletes in need to increase the energy available for exercise and delay the onset of fatigue [14,16]. Therefore, L-carnitine can help in this respect by increasing the beta-oxidation of fatty acids and sparing muscle glycogen for endurance athletes [16,17].

L-carnitine is derived either from dietary meat and dairy products or from endogenous synthesis from methionine and lysine in the liver and kidney [18]. L-carnitine has a crucial function in transporting long-chain and medium-chain fatty acids across the mitochondrial membrane where fatty acids go through β-oxidation and yield to the acetyl-CoA formation, which is a substrate of the tricarboxylic acid cycle (TCA) [19]. L-carnitine also maintains the mitochondrial acetyl-CoA/CoASH ratio, which is an important regulator of flux through the pyruvate dehydrogenase complex in carbohydrate metabolism [20,21]. Lastly, L-carnitine can act as antioxidants by inhibiting free radical generation [22,23,24,25].

Carnipure^®^ Tartrate (CT) is Lonza’s L-carnitine salt, specially formulated for lower hygroscopicity. The effects of CT on both exercise endurance and recovery have been previously reported [16]. The minimal dose of L-carnitine, however, has not been determined. Most of the studies conducted used high daily uptake (1000–4000 mg/d) of L-carnitine supplementation in athletes due to the difficulty in obtaining a clear effect of L-carnitine with lower doses [26,27,28,29]. The objective of this work is to investigate the effects of incremental doses of CT supplementation to the diet of rats in addition to an endurance exercise regimen. Effects on performance and fatigue by measuring time to exhaustion, recovery after exercise by measuring lactic acid, myoglobin, and malondialdehyde (MDA) levels, antioxidant capacity by measuring enzymatic activity (superoxide dismutase, SOD; catalase, CAT; glutathioneperoxidase; GSHPx), and other metabolite concentrations such as glucose and lipids, have been evaluated. Bodyweight changes, body mass index (BMI), and body composition were also assessed. To link these effects to the CT supplementation, plasma levels of L-carnitine and acetyl-carnitine were also determined.

## 2. Material and Methods

### 2.1. Animals

Male Wistar rats (*n* = 56, 8-weeks-old) were purchased from the Firat University Laboratory Animal Research Center (Elazig, Turkey). The animals were reared at the temperature of 22 ± 2 °C, the humidity of 55 ± 5%, and in a 12-h light–12-h dark cycle. The animals were fed a regular diet (Table 1) and water ad libitum. All animal procedures were approved by the Animal Experimentation Ethics Committee of Firat University (Elazig, Turkey) (2019/140--207). All procedures involving rats were conducted in strict compliance with the relevant laws, the Animal Welfare Act, Public Health Services Policy, and guidelines established by the Institutional Animal Care and Use Committee of the Institute.

### 2.2. Experimental Design

After a week of the adaptation period, the rats were divided into eight equal groups containing seven rats each. The initial body weights of the rats were similar among groups (214.53 ± 4.67, *p* > 0.05). The rats were fed either (1): a standard diet (Table 1) as a control with no exercise or Carnipure^®^ Tartrate (CT) supplementation at all (C0) or (2): a control diet supplemented with 200 mg/kg CT with no exercise (C200) or (3): a control diet supplemented with no CT but with exercise (*E*) or (4): a control diet supplemented with 25 mg/kg CT with exercise (E+C25) or (5): a control diet supplemented with 50 mg/kg CT with exercise (E+C50) or (6): a control diet supplemented with 100 mg/kg CT with exercise (E+C100) or (7): a control diet supplemented with 200 mg/kg CT with exercise (E+C200) or (8): a control diet supplemented with 400 mg/kg CT with exercise (E+C400). Table 1 shows the composition of the control diet fed to the rats. CT is a special quality of L-carnitine, which is produced via chemical synthesis. Lonza Consumer Health Inc., Morristown, NJ, USA provided CT. CT and placebo (physiological saline) were administered orally via gavage every day before exercise during the experimental period (6 weeks).

The rats were subjected to treadmill exercise on a motorized rodent treadmill (Commat Limited, Ankara, Turkey). The treadmill contained a stimulus grid at the back end of the treadmill, which gave an electric shock when the animal placed its paw on the grid. The apparatus had a 5-lane animal exerciser utilizing a single belt unit divided with walls suspended over the tread surface. To eliminate the diurnal variations, all exercise tests were applied during the same time of the day. A week of adaptation was provided as pre-training practice for the animals to get familiar with the treadmill equipment and handling. In doing so, the rats in the exercise training groups were accustomed to treadmill exercise over 5 days such that: (i) 1st day, 10 m/min for 10 min, (ii) 2nd day, 20 m/min for 10 min, (iii) 3rd day, 25 m/min for 10 min, (iv) 4th day, 25 m/min for 20 min, and (v) 5th day, 25 m/min for 30 min. Upon an adaptation of a week toward the treadmill system for the novel and stress impacts, the rats in treadmill exercise groups ran on the treadmill at 25 m/min for 45 min/per day and 5 days per week for 6 weeks, according to the protocol described by Sahin et al. [5]. The exercise model was chosen because the exercise model is the most common procedure to carry out animal exercise training from weeks to months at 45 min–1 h/day and 5 days/week. In addition, the exercise model provides adaptations to the cardiovascular system including physiological remodeling of the heart representative with increased O2 consumption, improvement of cardiac contractile function, and calcium handling [30]. The chosen long-term animal exercise model fits an effective program to benefit both healthy subjects and individuals with cardiovascular risk [31].

At the end of each exercise session, exhaustion time and average distance run were recorded. Endurance capacity was measured using treadmill running to fatigue on the last day of week 6 by compelling the rats to run on a motorized treadmill. This process is defined as the inability of the rat to maintain an appropriate pace despite continuous hand prodding for 1 min. After this application, the rats were removed from the treadmill. The rats were deemed to be fatigued when it was no longer able to continue to run on the treadmill as judged by the rat spending >50% of the time or >30 consecutive seconds on the electrical stimulus. 

Body weights were measured in rats using a scale after 6 weeks of exercise. The body mass index (BMI) was then calculated based on the formula: BMI (g/cm^2^) = body weight (g)/length (cm)^2^.

At the end of the experiment, all rats were subjected to overnight fasting and blood samples were taken from decapitated animals via cervical dislocation via anesthesia. In addition, visceral fat was carefully removed from the carcass and weighed. This procedure was carried out immediately after the last exercise session. Blood samples, collected by gel biochemical tubes, and serum samples were taken and centrifuged at 4 °C at 3000× *g* for 10 min in a chilled centrifuge. In addition, the tissues obtained from the animals were stored in a deep freeze at −80 °C until analysis.

### 2.3. Biochemical Analysis

Serum concentrations of glucose, aspartate aminotransferase (AST), alanine aminotransferase (ALT), urea, creatinine, total cholesterol, and triglyceride were assayed using a portable automated chemistry analyzer (Samsung LABGEO PT10, Samsung Electronics Co., Suwon, Korea). The rat lactate assay kit (Cayman Chemical Co., Ann Arbor, MI, USA) was used to measure the serum lactate concentrations through enzyme-linked immunosorbent assays (ELISA, Elx-800, Bio-Tek Instruments Inc., Winooski, VT, USA). ELISA (MyBioSource, San Diego, CA, USA) was also used in measuring serum myoglobin concentrations. The intra-and inter-assay coefficients of variation for lactate and myoglobin kits were both <13%. An enzymatic cycling method using commercial kits (MyBioSource, San Diego, CA, USA) was applied in assaying serum total and free L-carnitine concentrations. Acyl-carnitine was then calculated as subtracting free L-carnitine from the total L-carnitine amounts. A parameter of the oxidative stress indicator includes MDA concentrations assayed by a fully automatic High-performance liquid chromatography (HPLC, Shimadzu, Kyoto, Japan) equipped with a pump (LC-20AD), an ultraviolet-visible detector (SPD-20A), an inertsil ODS-3 C_18_ column (250 × 4.6 mm, 5 m), a column oven (CTO-10ASVP), an autosampler (SIL-20A), and a degasser. The activities of superoxide dismutase (SOD), catalase (CAT), and glutathione peroxidase (GSH-Px) were determined using the commercially available kits (Cayman Chemical, Ann Arbor, MI, USA), according to the manufacturer’s procedure.

### 2.4. Statistical Analysis

The data were statistically analyzed by one-way ANOVA using the SPSS statistical program (IBM, SPPS Version 21; Armonk, New York: IBM Corp). Differences between the groups were achieved by Tukey’s correction to control for false positives, which were generated by the multiple comparisons performed in the analyses. In addition, *p* < 0.05 was considered statistically significant. Data were reported as mean and standard deviations in tables. The data in the figures are presented as the median, minimum, and maximum values.

## 3. Results and Discussion

### 3.1. Effects on Exercise Performance, Recovery, and Fatigue

**Figure 1:**
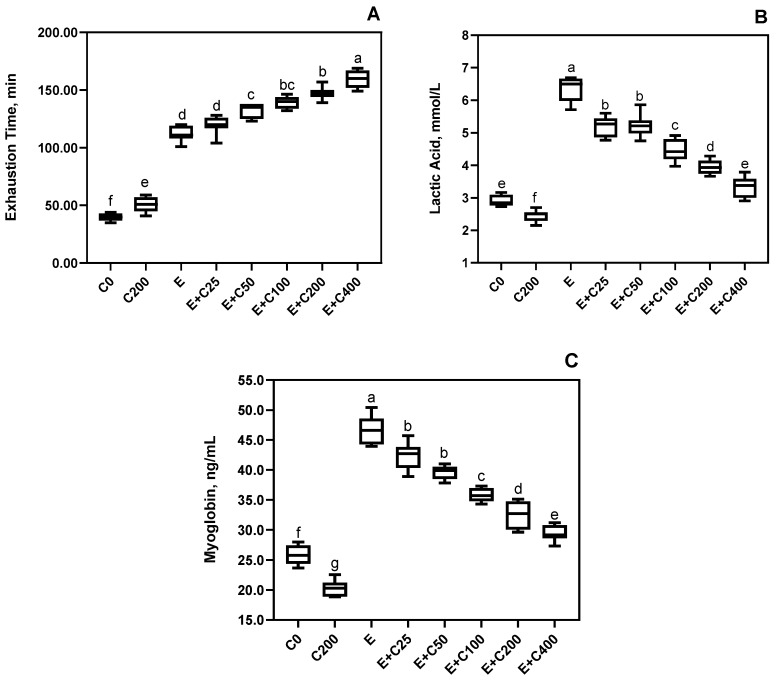
Effects of Carnipure tartrate supplementation on recovery parameters. The effects of Carnipure^®^ tartrate on the exercise performance (**A**), lactic acid (**B**), and myoglobin (**C**) levels in exercised rats but also in non-exercised rats (groups C0 and C200). Statistical comparisons are indicated with a different superscript (a–g) in the plots (*p* < 0.05, ANOVA, and Turkey’s post-hoc test). The data in the box and whiskers graphics are presented as the median, minimum, and maximum values for each plot.

Rats supplemented with 200 mg/kg CT and without exercise had greater exhaustion time when compared to rats without exercise nor supplementation (*p* < 0.05, Figure 1). However, increasing CT doses with exercise increased exhaustion time in a significant and dose-response manner started at 25 mg/kg of CT with a maximum at 400 mg/kg (Figure 1). It has been reported that L-carnitine supplementation prolonged exhaustion time in athletes [24,32,33]. However, no effects of L-carnitine supplementation on exercise performance were observed in male subjects or athletes [28,34,35,36]. The apparent controversial in the results may be explained by gender variability in humans, L-carnitine plasma bioavailability, and the type of exercise used. In humans, 1–6 g of L-carnitine taken orally led to only 5–15% [37,38]. Part of the low reported bioavailability may be due to higher renal clearance [39].

Exercise alone without CT supplementation had greater serum lactic acid and myoglobin concentrations compared to all other treatment groups (*p* < 0.05, Figure 1). However, both parameters decreased with increasing CT supplementation in exercised rats, which suggests a dose-response of these effects. Lactate production has been linked to insufficient muscle oxygenation and, consequently, results from fatigue and muscle soreness [40,41]. As outlined by other researchers, and in agreement with the present, L-carnitine has been shown to reduce lactate production and improve exercise performance [42,43,44,45]. However, other works reported no effects of L-carnitine on lactate production with exercise [27,46]. Exercise causes muscular damage and the leakage of proteins such as myoglobin into the circulation [47], as seen in the present work (Figure 1). Similar to the results of the present work, Naclerio et al. [48] reported attenuated fatigue and lower myoglobin plasma concentrations in soccer players supplemented with a multi-ingredient supplement including 1.5 gr L-carnitine-L-tartrate (Carnipure^®^ Tartrate). Both lactic acid and myoglobin, which are markers for muscle recovery after exercise, have been improved by CT supplementation in a dose-dependent manner (Figure 1).

### 3.2. Effects of Carnipure^®^ Tartrate Supplementation on Body Weight and Composition

**Figure 2:**
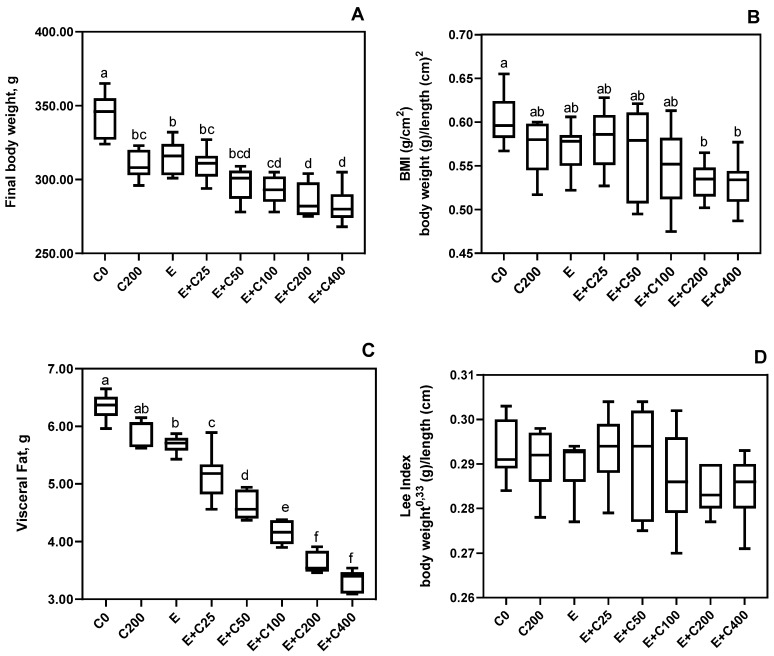
Effects of Carnipure Tartrate supplementation on body weight and composition. The effects of Carnipure^®^ tartrate on the body weight (**A**), body mass index (**B**) visceral fat (**C**), and Lee index (**D**) in exercised rats but also in non-exercised rats (groups C0 and C200). Statistical comparisons are indicated with different superscripts (a–g) in the plots (*p* < 0.05, ANOVA, and Tukey’s post-hoc test). The data in the box and whiskers graphics are presented as the median, minimum, and maximum values for each plot.

Although initial body weights of the rats were similar among treatments (*p* > 0.05), final body weights showed a dose-response decrease with exercise and CT supplementation. These effects were significant at 200 and 400 mg/kg doses (*p* < 0.05, Figure 2). Even rats fed a diet supplemented with 200 mg/kg CT with no exercise gained less body weight when compared with the control rats with no supplements or exercise (C0), which indicated that CT is effective in reducing BW gain even without exercise, to levels similar to that of rats going through exercise only. Similar responses were observed with visceral fat, which suggested that BW reduction may be due partly to a reduction in body fat driven by CT supplementation (200 mg/kg) even without exercise (Figure 1), which was recently reviewed by Talenezhad et al. [49]. L-carnitine supplementation to adults has an impact on body weight reduction as well as BMI and body fat, particularly for overweight and obese individuals. Several mechanisms have been suggested for L-carnitine being effective in body weight reduction. One of the suggested mechanisms is the fact that carnitine acyltransferases enzymes are a part of energy homeostasis and fat metabolism in the mitochondria, endoplasmic reticulum, and peroxisome [50]. The second mechanism is also related to the energy metabolism involvement of L-carnitine through induction of PPAR-γ expression, which reduces fatty acid synthesis [51].

Body mass index is used to evaluate body fatness. In accordance with the results observed for visceral fat, a decrease in BMI was obtained in exercising rats supplemented with CT in a dose-response manner. These effects are significant at 200 and 400 mg/kg doses (*p* < 0.05, Figure 1). Others reported that human subjects supplemented with L-carnitine lost more weight and had lower BMI when compared to subjects not receiving L-carnitine [52,53,54,55]. As shown by the results of the present work, exercise alone without supplementation caused a significant decrease in BMI when compared to control rats (C0) (Figure 1). Exercise, by increasing total energy expenditure, may decrease body weight, body fat, and BMI [56]. Similarly, L-carnitine supplementation resulted in weight loss by increasing energy expenditure with an effect on glucose and lipid metabolism [15]. Therefore, a combination of exercise and CT supplementation, as reported in the present study, may have additive/synergistic effects significant at 200 and 400 mg/kg doses.

Exercise alone or exercise combined with CT supplementation did not change the Lee index (Figure 1). The Lee index is a predictor for the risk of a cardiac event in patients undergoing noncardiac surgery.

### 3.3. Antioxidant Capacity

MDA, which is a marker for oxidative stress, had its serum concentrations decreased in exercising rats when compared to the control (C0) (*p* < 0.05, Table 2). However, increasing doses of CT supplementation in addition to exercise further decreased the serum MDA concentrations in parallel to increased antioxidant capacity, as shown by an increase in oxidative enzyme activities (Table 2). Parallel to the results of the present work, reduced MDA serum concentrations upon L-carnitine supplementation in rats with exercise [57], and in healthy exercising men [58] were also reported. Exercise is known to induce oxidative stress by increasing the generation of the reactive oxygen species (ROS), which is followed by lipid peroxidation and protein oxidation [59]. L-carnitine supplementation has been shown to promote the antioxidant enzyme activities of SOD, CAT, GPx, and glutathione (GSH) levels and to lower the MDA concentration, likely by its free radical scavenging and antioxidant properties, which, consequently, protects oxidative organ injury of kidney and heart dysfunction [60], commonly seen with aging [61].

### 3.4. Effects on Glucose and Lipid Levels

Serum glucose, triglycerides, and total cholesterol concentrations decreased in rats with exercise and without supplement or in rats supplemented with 200 mg/kg of CT and without exercise, as compared to control rats (C0, *p* < 0.05, Table 3). CT supplementation when added to exercise further decreased these parameters in a dose-dependent way with the greatest effect at 400 mg/kg. Pala et al. [57] reported an increase in glucose transporter mRNA expressions as well as a decrease in serum concentrations of triglyceride and total cholesterol in rats supplemented with L-carnitine and undergoing chronic exercise. A positive effect of L-carnitine supplementation on insulin-stimulated glucose disposal has been also proposed [62]. Muoio et al. [63] reported that supplementation with L-carnitine improved insulin resistance. Exercise alone decreased the serum glucose concentrations in rats (E) when compared to control rats (C0, Table 3). Exercise is considered the most prominent stimulus in increasing gene expression of skeletal muscle through GLUT4 transporter modulation [64].

Supplemental L-carnitine has been reported to shift the liver metabolism toward lowering the esterification and synthesis of triglycerides and very low-density lipoproteins (VLDL) cholesterol, while channeling mitochondrial β-oxidation of fatty acids [65,66,67]. Similar to the results of the present work, other researchers found reduced serum cholesterol, triglycerides, and free fatty acids, and increased HDL cholesterol upon L-carnitine supplementation [65,66,67,68,69]. However, the serum lipids, including triglycerides, total cholesterol, and low-density lipoproteins LDL cholesterol, were reported to remain unchanged with L-carnitine supplementation in other studies [70,71]. Contradicted results on lipid parameters of various human diseases upon L-carnitine supplementation have also been reported, suggesting underlying conditions that can complicate the interpretation [72].

### 3.5. L-Carnitine Plasma Levels

**Figure 3:**
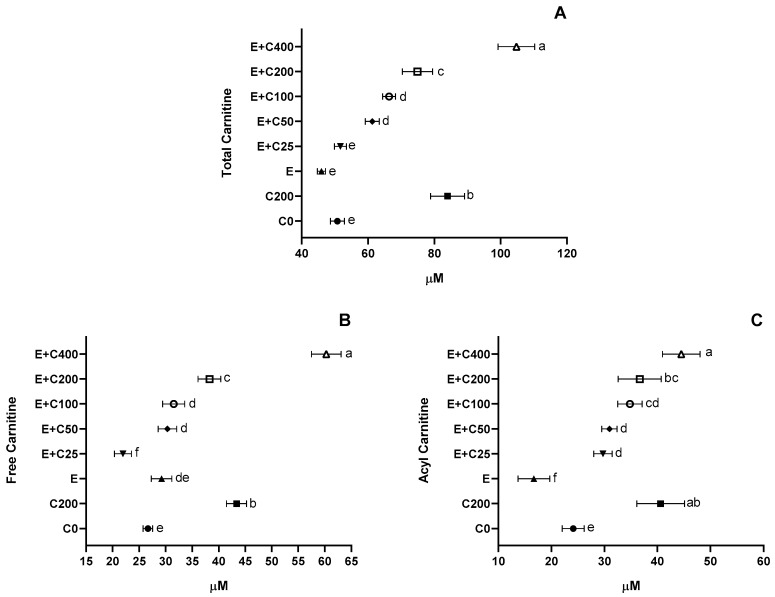
Effects of Carnipure tartrate supplementation on L-carnitine plasma levels. The effects of Carnipure^®^ tartrate on the total (**A**) free (**B**) and acyl (**C**) carnitine levels in exercised rats but also in non-exercised rats (groups C0 and C200). Statistical comparisons are indicated with a different superscript (a-g) in the plots (*p* < 0.05, ANOVA, and Tukey’s post-hoc test). The data in the box and whiskers graphics are presented as the median, minimum, and maximum values for each plot.

To link the observed efficacy effects to CT supplementation, serum plasma levels of total, free, and acylcarnitine have been assessed and showed a significant increase with CT by increasing doses with the greatest at 400 mg/kg doses (*p* < 0.05, Figure 3). Similarly, Bucioli et al. [73] also reported increased plasma L-carnitine concentrations in exercising rats that were exercised and supplemented with L-carnitine.

CT was also found to be safe based on liver enzyme measurements (Table 4). Based on the liver enzyme activities in Table 4, CT at doses up to 400 mg/kg (Human equivalent 3.87 g) was found to be safe. This finding is in line with authoritative safety assessments concluding that 2 g L-carnitine or 3 g L-carnitine L-tartrate are considered safe for humans (EFSA, Norway, etc.).

## 4. Conclusions

Results from the present work suggest that Carnipure^®^ Tartrate supplementation can be recommended to those who exercise for athletic performance as well as to the general population seeking to lower their body weight, BMI, and fat content. The observed effect on performance, fatigue, and recovery, as shown by a significant increase in time to exhaustion and reduction in markers of recovery such as myoglobin, lactic acid, and MDA, at doses as low as 25 mg/kg in rats, makes this supplement appealing to active individuals in general. In addition, Carnipure^®^ Tartrate supplementation helped reduce glucose and lipid levels, which are two metabolic parameters important for the general population of all ages.

The results of the present work revealed that CT supplementation alone even without exercise have a beneficial effect on combating overweight or obesity factors and related metabolic disorders. However, CT supplementation, particularly with higher doses when combined with exercise, have an even greater beneficial effect on health as well as busting athletic performance. The results of the present work also revealed that exercise with no dietary supplement provides similar health benefits, but not as much as when combined with dietary supplements. Exercise is crucial for a healthy life.

## Figures and Tables

**Table 1:** Ingredient and nutrient composition of the basal diet fed to the rats.

Ingredient	%
Maize	26.00
Wheat	14.00
Vegetable oil	3.00
Soybean meal, 48% CP ^1^	33.10
Sunflower meal, 30% CP	8.00
Wheat bran	7.00
Molasses	5.00
Limestone	0.80
Salt	0.80
DL-Methionine	0.80
Dicalcium phosphate	1.20
Vitamin and mineral premix ^2^	0.30
Analysis (%)	
Crude protein	24.27
Ether extract	4.55
Crude cellulose	4.04
Ash	6.91
Ca	0.75
P	0.41

^1^ CP: Crude Protein. ^2^ The vitamin-mineral premix provides the following (per kg): all-trans-retinyl acetate, 1.8 mg, cholecalciferol, 0.025 mg, all-rac-a-tocopherol acetate, 12.5 mg; menadione (menadione sodium bisulfate), 1.1 mg, riboflavin, 4.4 mg, thiamine (thiamine mononitrate), 1.1 mg, vitamin B-6, 2.2 mg, niacin, 35 mg, Ca-pantothenate, 10 mg, vitamin B-12, 0.02 mg, folic acid, 0.55 mg, d-biotin, 0.1 mg. manganese (from manganese oxide), 40 mg, iron (from iron sulfate), 12.5 mg, zinc (from zinc oxide), 25 mg, copper (from copper sulfate), 3.5 mg, iodine (from potassium iodide), 0.3 mg, selenium (from sodium selenite), 0.15 mg; choline chloride, 175 mg.

**Table 2:** Antioxidant capacity.

Items	Groups							
	Control	Car200	Exercise (E)	E+Car25	E+Car50	E+Car100	E+Car200	E+Car400
MDA, µmol/L	0.873 ± 0.017 ^a^	0.693 ± 0.021 ^b^	0.768 ± 0.015^b^	0.599 ± 0.021 ^c^	0.549 ± 0.014 ^c,d^	0.553 ± 0.018 ^c,d^	0.489 ± 0.019 ^d,e^	0.419 ± 0.009 ^e^
SOD, U/mg protein	83.88 ± 2.21 ^e^	95.87 ± 2.47 ^c,d^	85.21 ± 2.99 ^d,e^	96.49 ± 0.78 ^c,d^	102.89 ± 2.69 ^b,c^	109.68 ± 2.49 ^a,b^	114.56 ± 2.70 ^a^	120.16 ± 3.58 ^a^
CAT, U/mg protein	135.51 ± 2.61 ^e^	147.19 ± 2.01 ^d,e^	138.47 ± 3.69 ^e^	144.55 ± 3.21 ^d,e^	152.72 ± 2.50 ^c,d^	162.11 ± 1.97 ^b,c^	170.77 ± 2.57 ^b^	186.10 ± 2.50 ^a^
GSHPx, U/mg protein	36.87 ± 1.27 ^f^	48.74 ± 1.11 ^d^	42.56 ± 1.43 ^e^	49.75 ± 1.08 ^d^	55.91 ± 0.85 ^c^	59.57 ± 0.68 ^b,c^	62.58 ± 1.03 ^b^	69.06 ± 1.53 ^a^

MDA: malondialdehyde. SOD: superoxide dismutase. CAT: catalase. GSH-Px: glutathione peroxidase. Statistical comparisons are indicated with different superscript (a–f) in the same row (*p* < 0.05, *ANOVA and Tukey’s post-hoc test).

**Table 3:** Glucose and lipid levels.

Items	Groups							
	Control	Car200	Exercise (E)	E+Car25	E+Car50	E+Car100	E+Car200	E+Car400
GLU, mg/dL	115.29 ± 2.01 ^a^	109.43 ± 1.25 ^a,b^	104.43 ± 1.32 ^b,c^	101.14 ± 1.97 ^c^	103.43 ± 1.46 ^b,c^	101.43 ± 1.36 ^c^	98.43 ± 1.32 ^c,d^	92.43 ± 1.25 ^d^
TG, mg/dL	172.86 ± 4.08 ^a^	165.00 ± 2.32 ^a,b^	156.14 ± 4.08 ^b,c^	150.57 ± 3.21 ^c^	135.29 ± 2.54 ^d^	124.43 ± 1.46 ^d,e^	120.14 ± 2.11 ^e^	113.43 ± 1.39 ^e^
TC, mg/dL	149.57 ± 2.10 ^a^	142.62 ± 1.45 ^b^	138.22 ± 0.67 ^b,c^	135.10 ± 0.56 ^c^	127.57 ± 1.25 ^d^	115.08 ± 0.62 ^e^	110.23 ± 0.78 ^e^	102.27 ± 1.50 ^f^
BUN, mg/dL	21.59 ± 0.82	21.40 ± 0.65	20.67 ± 0.45	21.23 ± 0.90	21.34 ± 0.85	21.60 ± 0.82	21.31 ± 1.06	21.41 ± 0.69
TP, g/dL	6.50 ± 0.18	6.49 ± 0.13	6.50 ± 0.23	6.13 ± 0.43	5.93 ± 0.29	6.07 ± 0.44	6.37 ± 0.18	6.29 ± 0.20
TBIL, mg/dL	0.23 ± 0.01	0.24 ± 0.01	0.23 ± 0.01	0.22 ± 0.01	0.22 ± 0.01	0.23 ± 0.01	0.24 ± 0.01	0.23 ± 0.02

GLU: Glucose. TG: Triglyceride. TC: Total cholesterol. BUN: Blood urea nitrogen. TP: Total protein. TBIL: Total Bilirubin. Statistical comparisons are indicated with a different superscript (a–f) in the same row (*p* < 0.05, *ANOVA and Tukey’s post-hoc test).

**Table 4:** Safety parameters.

Items	Groups							
	Control	Car200	Exercise (E)	E+Car25	E+Car50	E+Car100	E+Car200	E+Car400
ALB, g/dL	3.40 ± 0.08	3.46 ± 0.05	3.47 ± 0.09	3.23 ± 0.16	3.09 ± 0.21	3.56 ± 0.05	3.39 ± 0.07	3.44 ± 0.06
GLOB, g/dL	3.04 ± 0.12 ^b^	3.10 ± 0.08 ^a,b^	3.41 ± 0.10 ^a,b^	3.27 ± 0.11 ^a,b^	3.29 ± 0.12 ^a,b^	3.53 ± 0.11 ^a^	3.11 ± 0.11 ^a,b^	3.16 ± 0.05 ^a,b^
ALT, U/L	103.43 ± 5.12	100.86 ± 4.07	99.43 ± 3.73	100.00 ± 4.62	101.00 ± 3.13	99.57 ± 4.49	104.86 ± 4.40	100.14 ± 2.96
AST, U/L	121.78 ± 6.73	122.94 ± 7.48	121.68 ± 3.64	121.31 ± 5.17	120.50 ± 3.77	122.57 ± 3.29	122.57 ± 3.72	121.57 ± 4.95

ALB: Albumin. GLOB: Globulin. ALT: Alanine aminotransferase. AST: Aspartate aminotransferase. ALP: Alkaline phosphatase. Statistical comparisons are indicated with different superscripts (a–b) in the same row (*p* < 0.05, *ANOVA, and Tukey’s post-hoc test).

## Abbreviations

CT	Carnipure^®^ Tartrate
MDA	Malondialdehyde
SOD	Super-oxide dismutase
CAT	Catalase
GSHPx	Glutathione peroxidase
TCA	Tricarboxylic acid cycle
BMI	Body mass index
AST	Aspartate aminotransferase
ALT	Alanine aminotransferase
ROS	Reactive oxygen species
